# Perspectives of the Teachers on Challenges and Possibilities to Online System of Education amid COVID-19 Outbreak in Balochistan, Pakistan

**DOI:** 10.1177/21582440231155063

**Published:** 2023-02-16

**Authors:** Rani Gul, Tehseen Tahir, Umbreen Ishfaq

**Affiliations:** 1 University off Malakand, Chakdara, Khyber-Pakhtunkhwa, Pakistan; 2 University of Haripur, Khyber-Pakhtunkhwa, Pakistan

**Keywords:** curriculum responsiveness, digital resources, online education, curriculum responsiveness, teachers preparedness

## Abstract

The study investigates the issues of access, quality, and other major challenges to the online system of education for students in Balochistan during this pandemic of Covid-19. Using the mixed-method design, 100 participants from schools and 7 curriculum experts responded to the survey and the interview questions. Survey results suggested that majority of the schools have enough digital devices, and teachers, to some extent, have skills to use technology in teaching, but limited electricity, funds, weak internet connections, and teacher training were some of the major challenges for them. In interviews, the participants showed disappointment in terms of responsiveness of the curriculum because the textbooks have manifold deficiencies in responding to online education. Considering these deficiencies, proposals were suggested to cope with the existing situation. The findings of the study call for the need on the part of the academia, educational stakeholders, and elected representatives to start joint efforts with national and international donor agencies, technology benefactors and telecommunication operators in developing digital infrastructure to provide students with access to education, quality, and lifelong learning through various pathways. The findings can be generalized to other underdeveloped regions both within and across the country, as the public schools are confronting the same issues and the online system of education has not yet been initiated.

## Introduction

When a natural disaster, a conflict, or a pandemic arises education is always halted first and resumed at the last (Gul & Khilji, 2021). COVID-19 pandemic has created a multidimensional and multilayered crisis across the world and has adversely affected all sectors. In mid-March 2020, to prevent the spread of the coronavirus, the government of Pakistan announced to close all private-public sector educational institutes without any anticipated re-opening date or future course of action. This closure of educational institutions directly affected 40 million school-going learners from pre-primary, primary to secondary, and higher secondary levels, besides the 22.7 million out-of-school children in the country (*Annual Status of Education Report*, 2019).

As the end of the pandemic remained unknown, to save students’ careers, the Higher Education Commission (HEC) of Pakistan immediately issued guidelines encouraging universities to switch to online education while taking into consideration their own respective capacities and infrastructure. Presently, in the higher education sector (universities and colleges), the online mode of learning has started but no efforts have been made by public schools in initiating an alternative system of education for the 40 million students enrolled in these schools across the country (United Nations Development Programme (UNDP) Human Development Report, 2020).

The report of Pakistan Social and Living Standards Measurement (PSLM) district-level survey 2019 to 20 revealed that from 2014–2015 to 2019–2020, literacy rate of Pakistan remained stagnant at 60%. The report further reveals that Punjab has the highest literacy rate of 64%, while Sindh has 58%, Khyber Pakhtunkhwa has 55%, and Balochistan has 46% (UNDP Human Development Report, 2020). With this rate, Pakistan is ranked at 152nd position out of 189 nations. Moreover, compared to other regional nations, Pakistan has not shown progress in the key educational matrix such as total enrolment ratio, educational budget and literacy rate. Pakistan’s literacy rate of 57% trails below its neighboring countries. The primary school dropout rate is 22.7% (behind Nepal and Bangladesh, the third highest in the region). During the 2018 to 2019 academic year, there were 522,400 serving teachers in 172,000 primary schools, while 438,100 serving teachers in 46,700 middle schools and 31,400 secondary schools. In this crisis, young and adolescent girls have been affected twice as much for they are confronted with multiple challenges to continue their education. The government has neither issued any guidelines nor has it introduced any alternate system of education and proper mechanism to provide teachers training, as students are sent home with no further guidance.

Owing to restricted access and quality of education, online schooling has been a potential replacement for formal education in Balochistan (Policy Analysis Report, 2018). The existing literature (Caldwell, 2006; Cavus et al., 2007; Figlio et al., 2010; Gul, Tahir, & Ishfaq, 2020; Peterson & Bond, 2004) affirms that online courses yield student results more comparable to those generated by face-to-face courses (Fanghanel et al., 2016). As Zhou et al. (2022), R. Gul et al. (2022) claimed that online education is more versatile, intimate, and competent. Online education is less costly with vast volumes of prepared content available all the time (Bukhari, Gul, et al., 2021). Students may no longer move to other countries to get better education, and can take instruction from the world’s top-notch professors and instructors, contributing to the socio-economic growth of the country (Bukhari et al., 2021; Gul, Zakir, et al., 2021). Skilled and well qualified educators will have more career options because of online education (Ahmad & Gul, 2021). This technology may turn a smartphone device into a classroom where students and teachers can have interactive teaching and learning opportunities with innovations and trends and, where the information can be revisited and easily accessible (Bukhari et al., 2021).

Online education may help alleviate the problem of subject instructors’ unavailability in schools (Ayub et al., 2021). Online education may increase research trends among students by giving opportunity of group projects and presentations. There may be no limitation on the number of seats in major courses in online programs and the majority of students can get higher education opportunities (Gul et al., 2021).

But such a quick shift in the education system might need a strategic plan to build upon the available resources and the major challenges these schools face which may hinder the shift to an online system of education. Therefore, the present study has investigated extent to which the existing infrastructure, access, quality issues related to curricula, teacher’s training and staffing, and other issues may pose challenges in initiating online system of education in Balochistan.

### Context of Education in Balochistan

Balochistan is the largest province of Pakistan by region. Unfortunately, the literacy rate in the province is just 46% (UNDP Human Development Report, 2020). According to the Economic Survey of Pakistan 2018 to 2019 report, among 3.6 million children, only 1.3 million attend schools. Moreover, most of these children (84%) attend public schools. These public schools are confronted with multiple issues regarding basic facilities, access, and quality of education for the past several years.

### Issues in Basic Facilities

In terms of “basic facilities,” schools have issues regarding access to power supply, potable water, well-equipped classrooms, shortage of teachers, availability of course books, lack of school boundaries and toilets, and furniture for students and teachers. Around half of the schools for girls have no toilets and boundary walls, while the conditions of boys’ schools are much worse. There are over 850 schools with no buildings. Constrains in infrastructure, availability and access to digital devices, together with inadequate internet facility to students residing in semi-urban and rural areas (Gul, 2018). Many toilets are not usable because water is not available and regular cleaning and maintenance is lacking. The fact that the schools are in such deteriorating conditions cannot compensate for an education awareness campaign.

### Issues in Access to Education

In terms of access, long distances between home and school, low population density, and a high number of single-room schools being run by a single teacher, limit access to education. Alif Ailaan 2018 report estimates that 13279 public schools are registered in Balochistan, among which 1,271 are elementary and 947 are secondary schools, while the rest are primary schools. The reports also revealed that on average, after every 30 km, there is a primary school; at every 260 km there is a secondary school.

The gender gap in education is persistent and deeply enmeshed with the varsity infrastructure challenge, especially for middle, high and higher secondary schools. Among the students attending government schools, girls barely make up 41% of the total strength. The situation gets worse in the rural areas of Balochistan. The government of Balochistan identified this gap as a major obstacle for girls’ education in these words:

School availability is further limited by ‘upward bottlenecks’ created by the drastic reduction in number of schools at the middle and secondary levels, leading to the exclusion of many children, especially girls (Balochistan Provincial Education Plan 2018, p. 14).

According to UNICEF (2018) report, the overall female literacy rate in the province is 26%. Due to security and mobility concerns, long-distance schools discourage parents from sending their girls. Here, the researcher states the story of a 14-year girl who left school after passing grade five because the nearest high school was a 10 to 15-min drive away.

“My parents want me to get an education,” she said, adding that if there was a school in the near area, they would let her start. But “I am not allowed to walk through the Bazar which is on my way to the secondary school,” she explained that her family consider this route unsafe and that they cannot afford to pay for her transportation cost. She hopes to come back to school: “This is my Day,” she said, “I awake, I read the Holy Quran and help my mother with housework”. She further said, “I request the government to upgrade our primary school to the high school or at least provide us with some alternate ways to get further education”.

### Issues in Quality of Education

In the domain of “quality,” curriculum, teachers, textbooks assessments, and environment of the school suffer from multiple issues. The poor pre-service training, lack of in-service training, and teachers’ absenteeism are the major problems that hamper the quality of the education system. In terms of content, duration, delivery, the quality of pre-service training programs is low, and their certificates/degrees do not comply with international standards (or even meet local needs). Lack of faculty in public sector training institutes and the malpractices, such as cheating in exams in the private sector, further undermine the quality of pre-service training programs. Besides, most of the in-service teachers are new to alternative contents of the textbooks and designing of the multimedia material to maintain students’ attention for an interactive teaching-learning environment. In this digital age, a 2-hr session might take up to 6 hr to capture and prepare. The goal of online education is not merely to give material, but also to keep students intellectually engaged (Gul & Khilji, 2021).

Similarly, for in-service teachers, a continuous professional development program based on an ongoing and holistic evaluation of the teacher’s students’ needs has never been organized. Data reveals that over 70% of in-service teachers have not undergone any kind of training. Due to fragile safety, security issues, and transportation problems, teachers face challenges to reach remote rural schools. Therefore, they mostly remain absent from duties. The posting of teachers does not depend upon the student ratio of 1 to 40 students, and there exists a large variance in teachers’ postings across and within districts.

After the 18th constitutional amendment in 2010, curriculum development has been decentralized to the provincial government of Balochistan. The curriculum standards are well-defined, but due to lack of expert staff in curriculum review or development process, the provincial government has implemented the Federal Government’s previous curriculum that was prepared in 2006. Now, over the years, implementing the curriculum is limited to textbooks only. Teachers, educational experts, and parents have neither been engaged in the curriculum development process, not have they seen the curriculum document. The teaching and assessment remain solely based on textbooks.

The textbooks do not reflect the interest of a child, as most are written in a dual narrative and their standards have never been established beyond basic input-based issues such as quality of the paper etc.

### Online Education in Balochistan Pre-COVID-19

Online education is a teaching-learning method that is based on the concepts of active pedagogy with distance learning characteristics. The online environment offers unprecedented opportunities for people who have limited access to education, as well as a new paradigm for educators in which dynamic courses of the highest quality can be developed (Das et al., 2019). Online learning in Pakistan has become more popularized in 2020 after the COVID-19 outbreak when all the educational institutes were prospered to shift to online modes of learning.

Before COVID-19, only the higher education sector was familiarized (to some extent) with the online mode of learning as universities across the country had a well-established Campus Management System (CMS) (Abida & Bilal, 2018), Learning Management System (LMS) facility and online digital library. Similarly, in this sector, many projects including Pakistan Education & Research Network (PERN), the Pakistan Research Repository (PRR) (Salameh et al., 2022), e-learning through Coursera and Massive Open Online Courses (MOOC) were launched to spread opportunities for digital learning among university students and providing the youth with the skills required to gain good employment options and economic empowerment.

On the contrary, no due attention was given to the school sector. At present, due to lack of pre-existing digital equipment, infrastructure and teachers’ capacities generally in Pakistan and particularly in Balochistan, the online/distant learning system is not functional. Even in the rest of the country, the phenomenon of online schooling is at the very grass root level.

## Research Questions

1. To what extent do technology infrastructure, access, and training pose a challenge to online learning in Balochistan?
2. To what extent do quality issues related to curricula and staffing pose a challenge to online learning in Balochistan
3. What are other major challenges facing Balochistan schools as they consider a shift to fully online education?

## Methodology

### Research Design

Mixed methods research was applied to understand the phenomenon under investigation. According to Ahmad, Gul, & Kashif, 2022) mixed-method research involves both quantitative and qualitative approaches to conduct a research study. Mixed methods research can create a thorough knowledge, explain the statistical results in further details and provide a more comprehensive picture than a purely quantitative or qualitative study (Ahmad, Gul, & Zeb, 2022). The design applied to this research was the explanatory methodology of sequential mixed methods (Quantitative → Qualitative = Explanation), which initially involves a quantitative survey and a qualitative follow-up interview with priority to quantitative intervention. The explanatory design was best suited for this study because the researcher wanted to understand the survey findings through a follow-up interview. Following this approach, quantitative data were collected first through a questionnaire and, as the initial picture emerged, the results were investigated using a qualitative approach. Qualitative data were collected later through individual interviews and analyzed using thematic analysis.

### Participants and Sampling

The target population for the study was principals and teachers serving in different public schools in rural and urban areas of Balochistan. The criteria for participation consisted of teachers’ vast teaching experience and engagement in instructional activities in the academic session 2020 to 2021. We received approval from our directorate of schools for human subject research before starting this work. Due to the lockdown, the quantitative survey was administered electronically to 68 cluster schools (one from the urban area and one from the rural area) in all 34 districts of Balochistan. The cluster schools are the high schools that supervise the curricular and co-curricular activities of all primary and elementary schools in that district. From each cluster school, two teachers and one school principal were randomly selected. The principals were contacted through phone and emails and were requested to participate in the study along with their teachers. The researcher received responses to the survey questionnaire from 38 schools in 26 different districts of the province. The respondents included 38 principals, 51 Secondary School Teachers (SST), 5 CT (certificate of teaching) teachers, and 6 subject specialists (SS) from these public schools. These districts included Quetta, Kohlu, Lehri, Pishin, Nushki, Kech (Turbat), Surab, Jhal Magsi, Killa Abdullah, Sibi, Loralai, Harnai, Awaran, Kalat, Barkhan, Killa Saifullah, Khuzdar, Mastung, Shinai, Zhob, Kharan, Musa Khel, Jaffarabad, Ziarat, Nasir Abad, and Lasbella.

Besides this, five principals and five SST teachers from sampled participants and seven curriculum experts from the curriculum wing, Quetta, were purposively selected and interviewed via phone to have more context and in-depth understanding of the opinions, experiences, and beliefs of educators and curriculum experts regarding curriculum responsiveness for the online system of education during this pandemic situation of COVID-19. The criterion for participation consisted of individuals’ adequate knowledge about the curriculum or experience in the curriculum development process and ability to detailed information that is generalizable. Quetta is the capital city of Balochistan, and the curriculum wing is located only in the capital city where experts from different areas of Balochistan are offering their services.

### Measures and Analytical Strategy

#### Survey Questions

A 10 questions survey questionnaire was developed according to the title and major theme of the study using Google forms with the help of the existing literature and surveys (Al-Oteawi, 2002; Isleem, 2003; Gulbahar & Guven, 2008; Sooknanan, 2014). The 10 questions survey reflects on three majors’ domains, including preparedness of the teachers, of the schools and of the curriculum.

Preparedness of the teacher’s domain included questions like, “How many teachers can create lessons using technology such as computer, internet etc.? What types of equipment/tools teachers use during their classes? What are the most popular technologies teachers use to teach students online and guide parents about the topics of different subjects?”

For Preparedness of the curriculum, the items in the survey included “Select all devices presently available in your school/ classroom which can help teachers to provide interactive audio/video instruction to students sitting at home. What delivery modes your school will use to assure that that lesson plans, videos, tutorials, and other resources are available for students at home?”

While for preparedness of the curriculum, items included in the survey were “The current curriculum and textbooks support independent learning. The current curriculum is responsive to sudden situations such as COVID-19. To what extent textbooks support online learning?” Two of the questions in the survey were on parents’ use and support of technology.

Detailed study of the existing literature on the questions understudy finds the research survey questions most comprehensive and suitable to pandemic situation. Before administration, the survey questionnaire was piloted. The Cronbach’s alpha for the 10 questions scale was found high (α = .87). The questionnaire was sent to curriculum experts for their critical pee-review to ensure both the face and content validity of the tool. The quantitative data collected from sample participants were analyzed with the help of SPSS using descriptive statistics.

#### Interview Questions

To get more in-depth information about the topic under investigation, the researchers formulated open-ended interview questions on major themes of the study, which included questions related to existing issues in the digital resources, different modes of delivery, teachers’ preparedness, and curriculum. Details of the questions are available in results section.

After analysis of the quantitative data, qualitative interviews were conducted on phone with the same questions used in the survey. The interviews were recorded after taking verbal consent from the participants. The recorded interviews were later transcribed and analyzed using the thematic analysis (Braun & Clarke, 2006) model. According to Braun and Clarke (2006), thematic analysis is “a tool to find, analyse and report the trends within data” (p. 79).

The researcher used Braun and Clarke’s (2006) six-step technique for this theme analysis. These six levels of data evaluation included: (1) familiarity, (2) coding, (3) generation, (4) theme searching, (5) theme naming and definition, (6) final reporting. This analysis technique is considered an excellent strategy in social science research due to its consistency and practicality. We reviewed, organized, and categorized participants’ comments, and the portions that were relevant to study topics were coded. Following the completion of this step, an internal assessment was performed in which one set of themes was developed from validation of two sets of themes. Each set of themes was then carefully analyzed, and overall code counts were calculated by counting each time a participant commented on a theme. The data analysis revealed the themes given in the results section.

## Results

### Demographic Information of the Respondents

In terms of the respondents’ professional role, most were secondary school teachers (51%) and principals (38%), although some were subject specialists (6%) and elementary school teachers had the designation of CT (certificate of teaching). The highest academic qualification of most of the participants (68%) was MA/MSc while, the second large bulk (18%) was observed with MS/MPhil degree holders. Besides, some respondents (13%) were BA/BSc /BS degree holders and one respondent academic qualification were FA/FSc (See Table 1).

**Table 1.** Descriptive Statistics of the Designation, Highest Academic, Highest Professional Qualification, and Experience in Teaching.

Position	Freq	Latest academic qualification	Freq	Latest prof. qualification	Freq	Years of experience	Freq
Head	38	MS/MPhil	18	MEd	72	5–10	46
SST	51	MA/MSc	68	BEd	19	11–15	24
CT	5	BA/BSc/BS	13	CT	1	16–20	13
SS	6	FA/FSc	1	Other	0	21–25	8
Other	0	Other	0			26–35	9

Similarly, for a professional qualification, the highest qualification of most of the participants (72%) was MEd Among other respondents, some (19%) had BEd degree and 1% had a CT certificate. Data also reveal that a great bulk of study respondents (46%) had 5–10 years of experience in their field. Besides, the other largest group of participants (24%) had experience of 11 to 15 years, 13% with 16 to 20 years of teaching experience, 9% with 26 to 35 years of teaching, and 8% with 21 to 25 years of teaching experience.

### Research Question 1: To What Extent Does Technology Infrastructure, Access, and Training Pose a Challenge to Online Learning in Balochistan?

To analyze this question, the participants’ responses on technology infrastructure, access and training are summarized in terms of frequencies and percentages and are presented in two sections. Section “A” deals with participants’ responses on technology infrastructure and access and section “B” deals with teachers’ training in technology.

#### Technology Infrastructure and Access Issues

When asked about top challenges for online learning (see AppendixTables A1 & A2), technology infrastructure and access were commonly cited. Ninety percent of respondents reported that teachers and students had not enough internet access at school or home to facilitate students in online education. Sixty-nine percent of respondents showed that in their schools, electrical supply was inadequate; while some participants (77%) stated lack of digital devices for audio/video presentations as a big hurdle in running an online program. Similarly, 40% of respondents mentioned that their schools had no computers, and 53% of respondents had no projector available at school. Only 34% of respondents had video recorder facility, 32% had audio recorders, and 13% had digital camera facility.

When asked about the mode of delivery to make the learning resources available for students online, majority of the respondents (82%) reported that they can record the lessons and send them through CDs and other flash drives to students’ homes while 76% reported that they can use social media as a major mode to deliver lesson plans, videos, tutorials, and other resources to students. Similarly, 29% of respondents were in the statement that they can use TV as a mode of instruction by providing the links and details of the programs offered for students, and 8% can use radio as a model for delivering learning resources, and some respondents (13%) stated that they cannot use any mode of delivery as they don’t have any digital gadgets available neither they have a proper light system at school. Most interviewees indicated that online learning and home-based learning may be among one of the alternate modalities for students during the COVID-19 epidemic. However, all the aforementioned modes face significant problems. Some participants, for example, stated that their districts had been without power for the last 2 months.

Others mentioned that there is a limited electric supply in some districts. Therefore, there would be huge hurdles in the way of online/e-learning. Apart from the access issues, some of the participants highlighted that online learning was only feasible with higher grades, where students could independently respond to the online lecture, screen-sharing, and assignments. The students of lower grades, like the primary could not be part of online learning without the teacher’s mentorship.

Participants supported online/e-learning on the condition that access concerns be resolved. One of the school participants expressed,

The online methods may help students for the continuity of their education in theCOVID-19 situation but there are heavy issues of access/support to online resources. For example, in our village district Ziarat, we do not have electricity supply for three months.

The participants anticipated that online learning will be influenced by two important factors: the degree of education of the parents, as well as the support structure and resources provided at home. This means that online learning requires essential equipment and conducive environment before it can be implemented. The attendees raised serious concerns about internet availability and connectivity.

The participants suggested these challenges could be overcome by identifying the important units/topics and delivering it to students through various channels like screen sharing, social media, Zoom, Skype, YouTube channels, or other methods. Students’ learning growth would not be hampered in this manner. They also stated that learning should not be restricted to textual texts. There may be more ways to educate youngsters using social learning ideas and they should learn material in the curriculum that specifically encourages independent learning on students’ part.

They further suggested that another option is to broadcast prepared courses via TV and radio stations. It is hoped that establishing some methods for students’ learning continuity would result in at least a 10% improvement. We feel that something is better than nothing. This favorable tendency ought to be introduced. We anticipate that the impact of establishing this healthy culture will compete with the multiplication effect of COVID-19.

### Teacher Technology Training/Comfort

To analyze the question regarding teachers’ technology training, the teachers were asked to rate their skills for being excellent, very good, good, fair, and not capable of the use of different digital equipment during teaching. School principals responses were also collected on teachers survey questionnaire as these principals also teach different courses to students. Table 2 presents the data in terms of percentages for each level.

**Table 2.** Level of Skill in Using Different Digital Gadgets.

I can use/operate this tool/equipment during my teaching	1. Excellent %	2. Very good %	3. Good %	4. Fair %	5. Not capable %
Digital camera	16	22	45	11	6
Smart phone	18	41	24	12	4
Projector	6	29	39	20	6
Audio recorder	17	43	32	05	4
Video recorder	20	38	30	7	4
Computer/laptop	18	42	22	13	4

Table 2 shows that majority of the respondents (45%) were good at using the digital camera, 22% of them were very good, while 16% were excellent at using the gadget. Similarly, 41% of respondents were very good, 18% were excellent and 24% were good at using smartphones during teaching. Besides, 39% of respondents marked themselves as being good at using projectors while 29% possessed very good skills, 6% excellent, and 20% were fair. Interestingly, most of the respondents (43%) rated themselves as very good, 32% as good, 17% as excellent, and 5% as fair in using an audio recorder. As for the skills on using a video recorder, surprisingly, 38% rated themselves very good and 30% marked good. We can also see in Table A1 that 20% of the respondents had rated themselves excellent and 7% fair in using this equipment. Similarly, in using computer/laptop 42% of the respondents rated themselves very good, 22% good 18% excellent, and 13% fair.

When asked about the level of skills in using the most popular technologies in teaching (see Table 3) majority of the respondents were confident in using Zoom and WhatsApp to teach students online and guide parents about topics. As we can see in Table 3 that for using Zoom 19% respondents rated themselves very confident while 47% confident, 30% less confident, and 33% not confident. While using WhatsApp 41% of respondent regarded themselves as confident and 21% as very confident, 18% less confident and 19% were not confident in using WhatsApp for teaching online. Besides, in case of google classroom, majority of the respondents (31%) rated less confident and 27% not confident while with a percentage of 29% and 12% respondents marked themselves as confident and very confident, respectively. Similarly, for Skype, majority of the respondents (54%) rated themselves as not confident, 27% less confident, 11% confident and 8% very confident, and in case of Microsoft Team majority of the respondents (82%) also marked themselves not confident while 12% less confident, and 6% confident. To use Facebook for online teaching, most of the respondents (43%) regarded themselves as less confident, 35% confident, and 13% reported being very confident. Similarly, for emails many respondents (41%) rated less confident, 40% not confident and 13% confident level while for using YouTube channel majority of the respondents (41) were not confident and 32% marked less confident in using it. However, 27% of respondents marked themselves as being confident. When teachers were asked about training on the use of technology in education, 63% of the respondents stated that they have received no proper training to use technology in online instruction.

**Table 3.** Level of Skills in Using the Most Popular Technologies in Teaching.

What are the most popular technologies you can use to teach students online and guide parents about the topics of different subjects?	1. Very confident %	2. Confident %	3. Less confident %	4. Not confident %
Use of Google classroom	12	29	31	27
Use of Zoom	19	47	30	33
Use of Microsoft Team	0	6	12	82
Use of Skype	8	11	27	54
Use of Wats App	21	41	18	19
Use of Facebook	13	35	43	9
Use of Emails	6	13	41	40
Use of YouTube Channel	0	27	32	41

In interviews, the participants reiterated that online education necessitates organized curricula, trained teachers, and a conducive learning environment for the required growth of the children. They complained that teachers have not been given due support and training. Teachers’ capacities have never been built for innovations in teaching. This scenario, however, might be managed by local teachers selected on a village, tehsil, and district basis. Local teachers maintain communication with parents and people of the community. The teacher may prepare lessons and home-based homework to compensate for the disturbance in study. A participant commented that, “The role of a local teacher is very important in online education. They can help students sequentially by teaching them the geography, history, culture, flora, and fauna of the vicinity. This would keep their learning context specific.”

The participant educators opined that there could be no learning without a teacher. The teacher’s absence from learning could also lead students to misunderstand the contents. One participant quoted his own story in the following way:

Can I share a small example? We have four 8-year-old children in our home. My younger brother has been trying to teach his children. I have also tried teaching my children to be an educationist. Children in our home start reading at an earlier age and grade level. When I asked my niece what her father had taught her. I was shocked to hear the misconception my brother has taught to his daughter.

The participants pointed out that learning without a teacher would be directionless learning. They believed the teacher as a guide who leads students to their destination. The participants suggested that issues of teachers training can be overcome by taking external expertise to develop the local teachers’ capacity. They proposed to develop a teacher handbook on natural or emergency crises as a major component of pre-service teachers’ training curriculum. They also urged the province government to establish pre-service teacher education standards and to tighten the regulatory framework for private sector schools. An overall continuous professional development approach should be designed, based on a holistic and continuing assessment of the requirements of instructors and students.

### Research Question 2: To What Extent Do Quality Issues Related to Curricula and Staffing Pose a Challenge to Online Learning in Balochistan

To analyze this question, participants were asked to rate their answer for being 1 “to a great extent,” 2 “to most extent,” 3 “to some extent,” 4 to “not at all,” and 5 to “any other.” The results are presented in terms of percentages in Table 4.

**Table 4.** Descriptive Statistics of the Responsiveness of the Curriculum and Textbooks.

Items on the scale	To a great extent	To most extent	To some extent	Not at all	Any other
The current curriculum and textbooks support independent learning	4	14	62	19	1
The current curriculum responds to sudden situations such as COVID-19	2	8	38	51	1
Textbooks don’t support online learning	6	40	30	22	2

Results (see Table 4) indicated that with the statement “textbooks don’t support online learning” most of the respondents (40%) agreed to most extent and 30% to some extent. While for the next statement “The current curriculum and textbooks support independent learning” majority of the respondents (62%) to some extent and 19% were with “not at all” opinion. But contrary to this, some participants (14% and 4%) agreed with “most to a great extent” that the current curriculum and textbooks support independent learning. Similarly, for the statement “The current curriculum responds to sudden situations such as COVID-19” most of the respondents (51%) did not agree and 38% of respondents agreed to some extent with this statement. But again, in contrast, some participants (8% and 2%) agreed from most to a great extent that curriculum responds to sudden situations such as COVID-19.

In interviews, the respondents were in the opinions that the curriculum has never been prepared for emergency scenarios such as COVID-19, particularly at the elementary grades. However, alternative modalities could be used for the continuity of students’ learning at the secondary level. One participant opined,

Besides COVID-19 pandemic, we could confront with more natural disasters such as earthquakes, floods, and wars which may cause such sudden closures of the educational institutes.

The participants pointed out that there could be some covert guidelines relating to such incidents, but the curriculum is mostly silent on this matter. Such material is not particularized and explicit. The participants highlighted this COVID as a test case for our school to design the curricula, making them configured with digital sources of learning. One participant claimed that,

Because our curriculum is pre-determined goals and outcome-based, we are not prepared for such emergent situations. The authorities have strict control over it. If the curriculum had been more adaptable, it would have been more responsive. The curriculum should be changed to generate analytical and critical thinkers. (Participant 9)

The participants reiterated that the curriculum is rigid and controlled and has manifold deficiencies in responding to both global and local needs at the same time. This situation has left the schooling non-responsive to both the local context and global needs. Aside from access concerns, several participants stated that online learning is only possible with higher grades, when students can reply to the online lecture, screen-sharing, and assignments autonomously. Students in lower grades, such as elementary, could not participate in online learning without the teacher’s guidance.

In response to the question whether the curriculum allows for independent learning, the participants said that there is no room in the curriculum for self-learning. The participants believed that the curriculum is politically influenced; therefore, it does not allow independent learning. These respondents’ opinions reflected that Participants were aware of the shortcomings of a preset and restricted curriculum. This predetermination robs instructors of their autonomy and reduces them to the role of information distributor. Participants agreed that both instructors and students should have some degree of autonomy so that the curricula could be redirected as per the contextual realities.

When the interviewees were inquired about staffing, they added that teacher’s recruitment and transfers are not based on merits. Teachers are recruited based on favoritism and even those people who barely passed their board examination in a second or third attempt are selected. Participants also added that less qualified (matriculation passed) untrained teachers are recruited by the provincial government and posted to different rural areas of Balochistan like Chagi, Jhall Magsi, Loralai, Harnai, where they rarely perform their duties and mostly take their salaries in the provincial capital of Quetta. One participant who also had worked on deputation as deputy district officer (DDO) for 3 years claimed that,

There is a great influence of politics in the teacher’s selection process. In the process of selection, candidates who have “good contacts” are given priority in selection.

The respondents also stated that teachers’ transfers are made due to personal enmities among teachers and district administrators. Such practices harshly affect those teachers who have been serving in that specific area for a long time and are best adjusted to the students and the local community.

To address these issues, the respondents suggested that “Must do” topics/themes should be identified from the curriculum. Special lessons on the highlighted topics/themes might be produced and disseminated with students via multiple channels–social media, screen sharing, YouTube channels, and other techniques. Students’ learning growth would not be hampered in this manner. Curriculum specialists can identify “must-know” ideas and share them with school officials. Authorities should train instructors to create lessons that may be shared with pupils. “Must know topics” should be identified both for instruction and assessment.

The respondents also added that there should be a separate curriculum ready for the time of emergencies, which may be a specialized curriculum to be used when things transcend normalcy. Or designed a portion in the curriculum as an emergency curriculum that guides them on how to face and get along with such situations. In case the course is designed, the assessment would also be designed for this emergent situation. Primary level classes do not need assessment. They further suggested that the best way to respond to such a situation is to prepare students as critical and analytical thinkers. This way, students, as future citizens, could deal with such challenges. The entire subject-curricula do not need to be taught. They should learn material in the curriculum that is specially designed for self-directed learning, particularly in the social sciences. Similarly, there could be a mapping of subjects as well. For example, subjects that could be learned as independent learning should be left out. Science, English, and Mathematics could be focused on. The participants further added that local teachers can play a pivotal role in being aware of the realities of the local context. She/he can immerse her/himself in creating opportunities for children, so that the children continue their learning. Currently, those teachers who are being posted in rural areas can facilitate students in their studies. However, this would need a staunch commitment from the top-down education hierarchy. They suggested that the directives could be issued to the cluster heads to assign responsibilities to teachers of her/his cluster. And teachers need to be incentivized by giving them some additional amount to motivate them for this task. They further stated that schools’ heads can play a role in this situation by inviting teachers to schools and directing them to map course content to be taught weekly, as per the relative situation. The research participants further suggested that teachers’ appointments should be made on a union council basis and priority should be given to highly qualified candidates so that in such situations the teachers may get easy access to the students and their parents in the local surrounding.

### Research Question 3: What Are Other Major Challenges Facing Balochistan Schools as They Consider a Shift to Fully Online Education?

When asked about the additional challenges, parental education, support, as well as teachers’ ability to communicate with parents were identified as major issues.

As we can see in Table 5 that majority of the respondents (F: 83) were of the opinion that only 25% to 50% of the students’ parents are educated enough to use technology. While other participants (F: 9) stated 50% to 75%, and five participants were in the perspective that 10% to 25% of the parents can use technology. Likewise, participants’ opinions on the ability of the parents to teach children at home depict that majority of the respondents (F: 55) stated that 10% to 25% of the parents can teach their children while the other bulk of respondents (F:34) believed that 25% to 50% parents can support children in their learning.

**Table 5.** Descriptive Statistics for Other Major Challenges.

Other Challenges	75%–100%	50%–75%	25%–50%	10–25%
Parents’ knowledge on the use of technology	3	9	83	5
How many parents do you think can teach children at home?	3	3	34	55

Table 5 reveals that for the majority of the respondents (33%), the major source of contact with parents was phone while 29% of the respondents kept contact through social media (Facebook and WhatsApp). Similarly, 14% of respondents reported face-to-face meetings and 10% reported letters as a major source. Similarly, 4% of the respondents used emails for communication while 10% of the respondents reported that they have no source of contact as the parents do not attend monthly meetings nor do they respond through phones and letters.

In interviews, the participants reflected that parent should be involved in the learning process of children. The government should prepare parents for the task before making them responsible for the learning of children at home as most of the parents are uneducated. However, the participant pointed out that,

This situation necessitates that the connection between school and parents be rebuilt and rejuvenated (Participant 4)

The participants revealed that parents should be engaged as supervisors to support students’ learning at home. Social media can be used to help parents to supervise their children in the time of emergencies such as COVID-19. Schools and teachers also need to remain in coordination with the parents through WhatsApp, Facebook and other platforms and provide them with complete guidelines for the learning contents.

## Discussion and conclusion

The study has investigated the issues of access, quality, and other major challenges for initiating an online system of education for students of Balochistan during this pandemic of COVID-19.

Analysis of the data collected through survey and interviews indicate that in terms of digital infrastructure and access, some schools are prepared to start online classes as they have enough devices and facilities to some extent which can help teachers provide interactive audio/video instruction and can use all the delivery modes including T.V, radio channels, and social media to assure that lesson plans, videos, tutorials, and other resources are available for students. However internet access, limited electric supply, funds availability, and lack of digital devices for audio/ video presentations are some of the major challenges for them. Studies have proven that without ensuring equity among students of all levels, technology integration cannot lead to efficient and effective learning (Gul et al., 2020). The findings are consistent with reports of UNESCO (2016); Policy Analysis Report, 2018 and Alif Ailaan, 2019 that Balochistan has always been under educational crises because of non-availability of very basic facilities in schools including boundary walls, net facility, portable water, electricity supply, and lack of teachers’ training at schools and colleges. Similarly, in an article Mehmood Bugti (2020) also pointed out that Balochistan, despite being Pakistan’s wealthiest province regarding natural resources, is failed to attract funds to its schools. Literature shows that the availability of basic facility especially electric supply, directly affects students’ learning. The recent survey data of World Bank (2018) and UNESCO (2016–2019) from 210 countries revealed that the availability of electricity has shown a great impact on students’ education as it facilitates teachers to use all digital educational devices. It has also been shown in the same report that electrifying schools and the availability of proper net connections create comfortable and successful schools that show better performance through student’s enrolment and high academic achievements. Students must have secure internet connections and other digital devices to get online education. We realized that anything we put into the world of digital education had to be of the highest quality, something that could connect, communicate, and interact with the stakeholders (Gul & Khilji, 2021).

Findings further reveal that teachers also have the skills to use available digital devices and to some extents are familiar with the latest software, including YouTube Channel, Google classroom, Zoom, Skype, and other social media, etc. which can help them in teaching online. But they have not received proper training on the use of technology for teaching and assessment purposes. Technical abilities also have a strong impact on the online teaching. Research has revealed that if teachers become better acquainted with technology (computers, internet, and media tools), they are more ready to teach online (Batool et al., 2022; Gul et al., 2022).

In terms of quality and staffing, the survey interview findings disclose that respondents were disappointed with the responsiveness of the curriculum for online education during the pandemic. The respondents opined that the predetermined and controlled curriculum is mostly silent in responding to the needs of the students and teachers in the online system of education because the textbook material is not particularized and explicit and has manifold deficiencies in responding to both local and global needs at the same time. Similarly, teachers’ postings, local teachers’ non-availability in rural areas, and parental support and the level of interaction with schools were also detected as a big challenge for online education. Children are frequently exposed to a violent home environment. This type of environment negatively influences children’s emotional and mental health which affects their academic performance (Ali et al., 2021). Considering these deficiencies, proposals were suggested by the respondents to cope with the existing situation.

Considering the findings and respondents’ suggestions, it is recommended that our educational stakeholders and elected representatives need to work with national and international donors for Pakistan who fund the largest education programs in the country and have played a key role in strengthening the education sector of the country. These agencies include the United States International Development Agency (USAID), United Nations Development Programme (UNDP), World Bank, Asian Development Bank, and the German Development Agency (GIZ). With the help of these donor agencies, the provincial and federal government may start a coordinated effort with technology benefactors and telecommunication operators in preparing missing facilities and replenishment of District Wise School Infrastructure Development Plans (DSIDPs) to guide need-based investment schemes. The study also recommends providing computer laboratories to all secondary schools, including facilities for digital teaching and assessment, providing all students with access to education, quality, and lifelong learning through various pathways. As in France, China, and UAE, 60 plus educational institutions, publishers, media organizers, and industry experts are providing computers and tablets along with subsidizing mobile data packages, printed assignments and other educational resources including books, evaluation, assessment tools, and videos to the students who do not have access to the Internet, a similar initiative is required in Balochistan.

The study also recommends developing the existing pre-service education programs that produce quality teachers well acquainted with content knowledge, digital pedagogical skills, and assessment tools. Likewise, in-service teacher development institutions should be strengthened to promote Continuous Professional Development (CPD) based on local needs and environment. A formal coordination mechanism should be developed between the Provincial Institute of Teacher Education (PITE) and the Directorate of Schools to ensure transparent selection of teachers, realistic needs assessment and feedback mechanism and enhance provincial capacity to develop, implement and review quality in-service teacher training. While teachers endeavor to find new methods to interact with their students via technology, we must pay close attention to what we are teaching to our students (Gul et al., 2020). The study recommends that courses must be based on students’ experiences and should encourage dynamic and versatile thinking, compassion, and tolerance among students. Teachers should be prepared to act as a facilitator. This shift from traditional teaching to online may provide ample opportunities for teachers for self-learning by studying from a range of platforms and support customized learning. The instructors should be trained as “competent” individuals who can go with students’ expectations (Gul et al., 2021).

It is further recommended to develop relevant, comprehensible, and transparent curricular targets to achieve learning outcomes, responsive to the current and emerging needs and challenges of the learners. The federal and provincial Bureau of Curriculum (BOC) and policymakers need to strengthen the capacity of the existing curriculum organizations, develop collaboration and strong linkages among them to improve the existing curriculum, provide quality textbooks/learning materials and better assessment practices. A mechanism for systematic and continuous curriculum improvement and a comprehensive curriculum implementation framework (CIF) needs to be developed and implemented at the provincial level. A proper mechanism should also be prepared at the district department of education to develop and ensure a strong relationship between teachers and parents. Schools should collaborate with existing educational institutions to encourage online learning through teacher development programs and exchange of free digital services until they are prepared for the challenges of online education. Schools should also provide parent facilitation services to provide parents with information and support in a timely and skillful manner.

In conclusion, even though many issues exist in initiating an online system of education, these are not inescapable. As change is not optional, it is time for academia to transform into an online system of education. Literature has affirmed that online teaching yields student results more comparable to those generated by face-to-face teaching (Caldwell, 2006; Figlio et al., 2010; McConnell & Graham, 2016; Mentzer et al., 2007; Tufail et al., 2022). If the government gets stuck in a vicious cycle where net connectivity and electric supply are denied him to initiate an online system of education, it will lead to widespread unemployment and catching pace with the rest of the world will remain an unfulfilled dream for the youth of Balochistan.

### Study Implications

Results of this study are specific to online schooling in Balochistan but the findings may be generalized to other provinces of Pakistan; as public schools in Pakistan are confronting the same issues, and online system of education for school students has not been initiated to date. Study implications are significant for higher-level studies and researchers of the national and international levels as well. The findings of the study may open new windows for a combined collaboration of the schools with the technologically developed institutes around. It may compel the institutes to compete with online education websites and the platforms offering free certified courses. The findings of the study also expect a long-term shift in the teaching-learning process which is expected to be in practice in post-Covid-19 era. This long-term shift in the teaching-learning process promotes the blended learning concept. The findings are also beneficial for developing countries whose demographic and economic conditions are like Pakistan, for example, India, Sri Lanka, and Bangladesh, etc. where the education sector specifically at the rural side is confronting similar issues as in Pakistan.

### Study Limitations

The study has some limitations. Firstly, the existing literature has insufficient available data on the topic under study. Therefore, we referred to results from few studies. Secondly, the study is limited to a single province and further study can be extended to other provinces of the country. Thirdly, the study is limited to the technical skills of the teachers in using digital resources. Further studies could examine teachers’ competencies in online teaching methodologies and assessments as the shift from conventional teaching to online teaching require good preparation of teachers to adapt to the new paradigm shift. Fourthly, this study is limited to online education at the school level, further studies can be conducted at a higher education level. Finally, the data are based on self-reported due to the nature of the study. Some teachers may not be familiar with all the competencies for online teaching, and there might be a response bias.

## Appendix

**Table A1.** Available Digital Resources in Schools.

Select all the devices presently available in your school/classroom which can help teachers to provide interactive audio/video instruction to students sitting at home.	Name of the device	*F*	%
	Internet devices	15	39.4
	Computers	28	73.6
	Projector	18	47.3
	Audio recorder	12	31.5
	Digital camera	05	13.1
	Video recorders	13	34.2

**Table A2.** Different Modes of Delivery School Can Use to Assure that Learning Resources are Available for Students Online.

What Modes of delivery your school can use to assure that lesson plans, videos, tutorials and other resources are available for students online.	Modes of delivery	*F*	%
	Social media	29	76
	Recorded lessons	31	82
	TV	11	29
	Radio	3	8
	Other:	5	13
